# The Impact of Bariatric Surgery on the Incidence of Colorectal Cancer in Patients with Obesity—a Systematic Review and Meta-analysis of Registry Data

**DOI:** 10.1007/s11695-023-06674-4

**Published:** 2023-06-21

**Authors:** Matthew G. Davey, Odhrán K. Ryan, Éanna J. Ryan, Noel E. Donlon, Ian S. Reynolds, Naomi M. Fearon, Sean T. Martin, Helen M. Heneghan

**Affiliations:** 1 Discipline of Surgery, The Lambe Institute for Translational Research, University of Galway, Galway, H91YR71 Ireland; 2 Surgical Professorial Unit, St. Vincent’s University Hospital, Elm Park, Dublin 4, D04 T6F4 Ireland; 3 Royal College of Surgeons in Ireland, 123 St. Stephens Green, Dublin 2, D02YN77 Ireland

**Keywords:** Bariatric surgery, Colorectal cancer, Meta-analysis, Obesity

## Abstract

**Purpose:**

Cancer and obesity represent two of the most significant global health concerns. The risk of malignancy, including colorectal cancer (CRC), increases with obesity. The aim of this study was to perform a systematic review and meta-analysis to determine the value of bariatric surgery in reducing CRC risk in patients with obesity using registry data.

**Materials and Methods:**

A systematic review and meta-analysis were performed as per PRISMA guidelines. The risk of CRC was expressed as a dichotomous variable and reported as odds ratios (OR) with 95% confidence intervals (CIs) using the Mantel-Haenszel method. A multi-treatment comparison was performed, examining the risk reduction associated with existing bariatric surgery techniques. Analysis was performed using RevMan, R packages, and Shiny.

**Results:**

Data from 11 registries including 6,214,682 patients with obesity were analyzed. Of these, 14.0% underwent bariatric surgery (872,499/6,214,682), and 86.0% did not undergo surgery (5,432,183/6,214,682). The mean age was 49.8 years, and mean follow-up was 5.1 years. In total, 0.6% of patients who underwent bariatric surgery developed CRC (4,843/872,499), as did 1.0% of unoperated patients with obesity (54,721/5,432,183). Patients with obesity who underwent bariatric surgery were less likely to develop CRC (OR: 0.53, 95% CI: 0.36–0.77, *P* < 0.001, *I*^2^ = 99%). Patients with obesity undergoing gastric bypass surgery (GB) (OR: 0.513, 95% CI: 0.336–0.818) and sleeve gastrectomy (SG) (OR: 0.484, 95% CI: 0.307–0.763) were less likely to develop CRC than unoperated patients.

**Conclusion:**

At a population level, bariatric surgery is associated with reduced CRC risk in patients with obesity. GB and SG are associated with the most significant reduction in CRC risk.

**PROSPERO Registration:**

CRD42022313280.

**Graphical Abstract:**

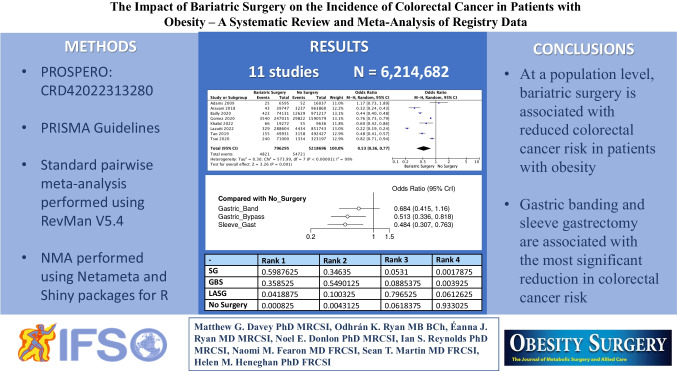

## Introduction

The incidence of obesity and cancer has been increasing in recent years, contributing to significant challenges to healthcare economies [1, 2]. The incidence of obesity has nearly doubled since 1980 [3], and the prevalence of obesity has recently reached “epidemic proportions” with estimations that 70% of the adult population are now overweight or have obesity [3–5]. Concurrently, the global cancer prevalence continues to increase [2]; therefore, the prioritization of cancer and obesity diagnosis and treatment is of the utmost importance to medicine and surgery in the twenty-first century [6].

Obesity-related cancers account for 11.9% and 13.1% of all cancer diagnoses made in male and female patients respectively [7]. The estimated obesity-related risk of colorectal cancer (CRC) has a dose-response relationship, increasing by 24% in male patients and 9% in female patients with every 5 kg/m^2^ increase in body mass index (BMI) [8]. Moreover, all measures of adiposity have been shown to increase the risk of CRC in people with obesity [8-12]. Notwithstanding, recent data from the older Swedish Obese Subjects (SOS) study illustrated similar CRC risk in patients with obesity at 22 years of follow-up, irrespective of whether they had undergone bariatric surgery, despite the inclusion of outdated bariatric procedures [13]. Therefore, clarifying the impact of severe obesity on cancer incidence remains a clinical priority to develop mitigation strategies and improve access to obesity treatments [14, 15].

Bariatric surgery has excellent outcomes for treating obesity and obesity-related complications [16], with several studies illustrating improvements in metabolic and cardiovascular diseases and long-term survival [11, 17, 18]. It may also be a promising strategy to reduce cancer incidence in people with obesity [19-21]. Recent recommendations from the American Society for Metabolic and Bariatric Surgery (ASMBS) Clinical Issues Committee highlight the value of bariatric surgery in mitigating obesity-related cancer risk [22], with CRC risk reduced in patients with obesity following bariatric surgery, as illustrated in several previous analyses [23, 24]. Regrettably, these studies failed to provide robust data that could be translatable to a population level. Therefore, the necessity to establish the “real-world” population risk of CRC in patients with obesity who have undergone bariatric surgery compared to their counterparts using large volume, registry data is imperative to ensure accurate and informative preoperative patient counseling.

Accordingly, the aim of the current study was to perform a systematic review and meta-analysis to determine the value of performing bariatric surgery in reducing CRC risk in patients with obesity using registry data only for application to “real-world” decision-making.

## Methods

### Materials and Methods

A systematic review was performed in accordance with the preferred reporting items for systematic reviews and meta-analyses (PRISMA) checklist and meta-analysis and systematic reviews of observational studies (MOOSE) guidelines [25, 26]. Local institutional ethical approval was not required for this study. This study was registered with the International Prospective Register of Systematic Reviews (PROSPERO: CRD42022313280).

#### Search Strategy

An electronic search was performed of the PUBMED, EMBASE, and Scopus databases on the 18th of February 2022 for relevant studies which would be suitable for inclusion in this study. The search was performed of all fields under the following headings: (bariatric surgeries [MeSH Terms]), (cancer, colorectal [MeSH Terms]), which were linked with the Boolean operator “AND.” Included studies were limited to those published in the English language. Included studies were not restricted based on the year of publication. All titles were initially screened, and studies deemed appropriate had their abstracts and full texts reviewed.

#### Inclusion and Exclusion Criteria

Studies meeting the following inclusion criteria were included: (1) Studies assessing the value of bariatric surgery to reduce CRC incidence in patients with obesity; (2) eligible studies had to use registry data in their analysis; and (3) studies had to include patients with obesity only. Studies meeting any of the following exclusion criteria were excluded from this study: (1) Studies not evaluating the impact of bariatric surgery on CRC risk; (2) studies including patients without obesity; or (3) studies not using registry data. Where overlapping registries were identified or suspected, the study representing the largest number of patients was included for analysis.

#### Data Extraction and Quality Assessment

The literature search was performed by two independent reviewers using a predesigned search strategy. Duplicate studies were manually removed. Each reviewer then reviewed the titles, abstracts, and/or full texts of the retrieved manuscripts to ensure all inclusion criteria were met before extracting the following data: (1) first author name, (2) year of publication, (3) study design, (4) country of origin, (5) registry database used, (6) years from which patients were included, (7) number of patients with obesity included, (8) number of patients with obesity included who underwent bariatric surgery, (9) number of patients with obesity included who did not undergo bariatric surgery, (10) number of patients with obesity who underwent bariatric surgery who subsequently developed CRC, and (11) number of patients with obesity included who did not undergo bariatric surgery who subsequently developed CRC. Data specific to patient outcomes and CRC incidence (expressed as hazards ratios (HR), 95% confidence intervals (95% CI), and *P* values) were directly extracted from tables and study text. HR and associated standard errors were calculated from Kaplan-Meier curves where relevant. In case of discrepancies in opinion between the reviewers, a third reviewer was asked to arbitrate.

#### Statistical Analysis

Clinicopathological characteristics and outcomes were recorded using descriptive statistics. The Fisher’s exact (†) and chi-square (*χ*^2^) tests were used as appropriate to determine the association between bariatric surgery among patients with obesity who subsequently developed CRC [27]. Thereafter, treatment and survival outcomes were expressed as dichotomous or binary outcomes, reported as odds ratios (ORs) and 95% confidence intervals (95% CIs) following estimation using the Mantel-Haenszel method. A meta-analysis of time-to-effect measures from each eligible study was also performed. Estimates of log hazard ratios (HRs) and their standard errors were combined using the generic inverse-variance method. Either fixed- or random-effects models were applied based on whether significant heterogeneity (*I*^2^ > 50%) existed between studies included in the analysis. Symmetry of funnel plots was used to assess publication bias. Statistical heterogeneity was determined using *I*^2^ statistics. Statistical significance was determined to be *P* < 0.050. Statistical analysis was performed using Review Manager (RevMan), Version 5.4 (Nordic Cochrane Centre, Copenhagen, Denmark).

Bayesian network meta-analyses (NMAs) were conducted using Netameta and Shiny packages for R to perform a multi-treatment comparison (MTC) of the effect of the available techniques in bariatric surgery [28]. Effect sizes were described with 95% credible intervals (CrIs). Rank probabilities were plotted against the possible ranks for all competing treatments. The confidence in estimates of the outcome was assessed using the “Confidence in Network Meta-Analysis” (CINeMA) [29]. Methodological and risk of bias assessment of the included studies was undertaken using the Newcastle Ottawa Risk of Bias Assessment tool for observational studies [30].

## Results

### Literature search

**Fig. 1:**
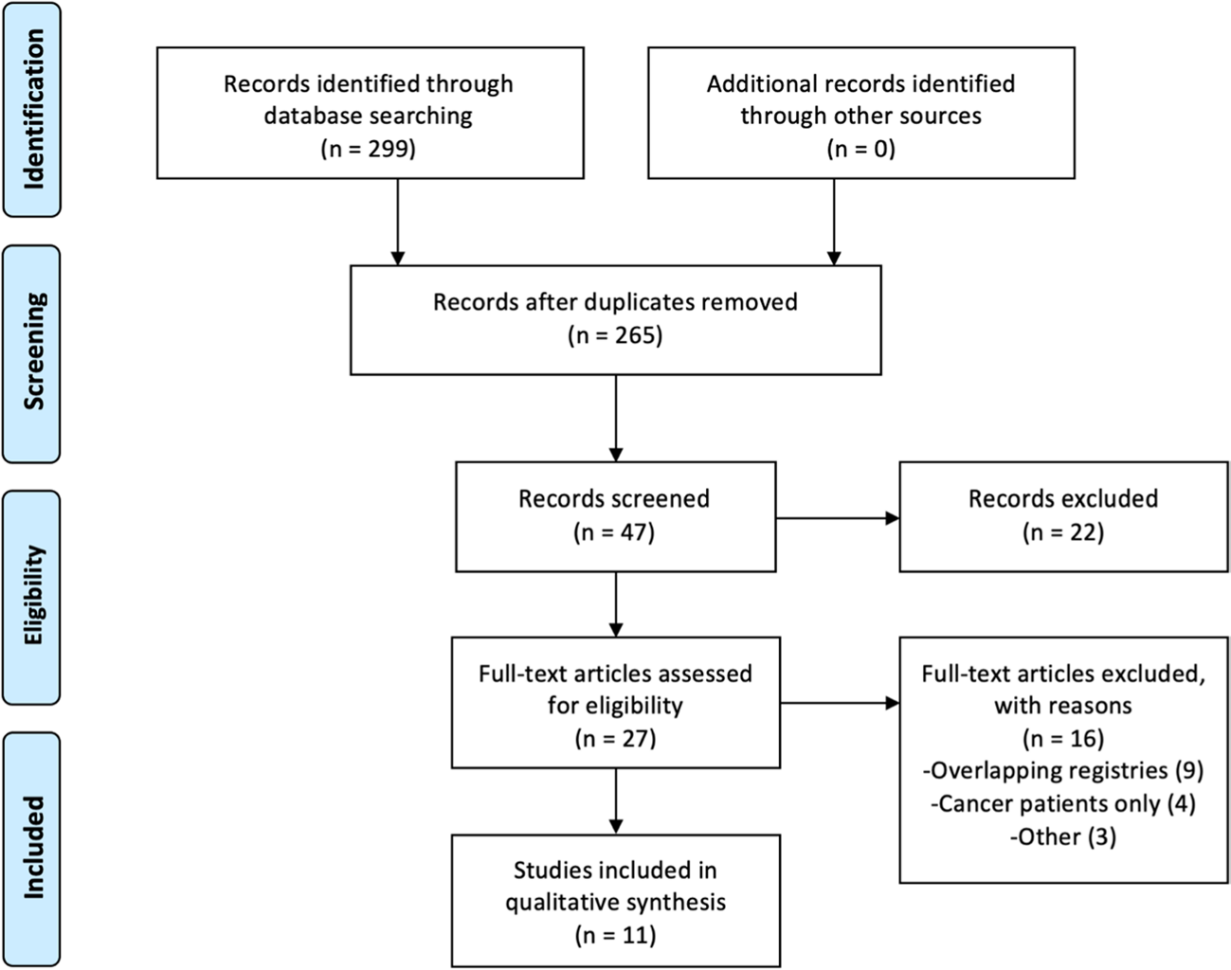
PRISMA flowchart illustrating the systemic search strategy

The systematic search strategy identified 299 studies, of which 34 duplicate studies were manually removed. The remaining 265 studies had their titles screened for relevance before 49 abstracts, and 27 full texts were reviewed for edibility. Eleven registry studies fulfilled the inclusion criteria and were included in this systematic review [31-41] (Fig. 1).

### Study Characteristics

Of the eleven included studies, six provided registry data in relation to CRC risk following bariatric surgery in the USA (6/11). All included studies were of retrospective design (100.0%, 11/11). Publication dates of included studies ranged from 2009 to 2022, representing patients from 1980 to 2018. Seven studies provided definitions of obesity, with three different definitions used in various registries. The breakdown of the type of bariatric surgery, either laparoscopic gastric bypass surgery (GB), laparoscopic adjustable gastric banding (LAGB), or laparoscopic sleeve gastrectomy (SG), was outlined in ten of the included studies. Data from the included 11 studies are summarized in Table 1.

**Table 1:** Details of the 11 included studies in this analysis

Author	Year	Study	Country	Registry	Years	Definition Obesity/Indications for Bariatric Surgery	Surgery Details	ROB
Adams	2009	RC	USA	Utah Cancer Registry	1984–2007	BMI >35kg/m^2^	GBS	Low
Aravani	2018	RC	UK	HES Database	1997–2013	-	Multiple	Low
Bailly	2020	RC	France	FNIIS	2009–2018	-	LAGB, SG, GBS	Low
Ciccioriccio	2021	RC	Italy	ISOS Database	2010–2015	-	SG, GBS	Low
Gomez	2020	RC	USA	NIS	2010–2014	BMI >35 kg/m^2^	Multiple	Low
Khalid	2022	RC	USA	All Payers Claims Database, Mariner-15	2010–2018	BMI >40 kg/m^2^ or BMI >35 kg/m^2^ and at least one obesity related comorbidity	SG, GBS	Low
Lazzati	2022	RC	France	National Discharge Database	2010–2017	BMI >40 kg/m^2^ or BMI >35 kg/m^2^ and at least one obesity related comorbidity	-	Low
Rustgi	2021	RC	USA	WHMSCCE Database	2007–2017	BMI >40 kg/m^2^ or BMI >35 kg/m^2^ and at least one obesity related comorbidity	Multiple	Some
Schauer	2019	RC	USA	Kaiser Permanente	2004–2014	BMI >35 kg/m2	Multiple	Low
Tao	2019	RC	Scandinavia	NOS Database	1980–2015	-	Multiple	Low
Tsui	2020	RC	USA	NYSPRCS Database	2006–2012	BMI >30 kg/m2	LAGB, SG, GBS	Some

*RC* retrospective cohort, *USA* United States of America, *UK* United Kingdom, *BMI* body mass index, *GBS* gastric bypass surgery, *SG* sleeve gastrectomy, *LAGB* laparoscopic gastric band, *HES* hospital episode statistics, *FNIIS* French National Insurance Information System, *ISOS* Italian Society of Obesity Surgery, *NIS* national inpatient sample, *WHMSCCE* Watson Health Market Scan Commercial Claims and Encounters, *NOS* Nordic Obesity Surgery, *NYSPRCS* New York State–wise Planning and Research Cooperative System, *ROB* risk of bias assessment performed in accordance with Newcastle Ottawa Scale

### Clinicopathological Characteristics

From the 11 independent registries, 6,214,682 patients with obesity were included. Of these, 14.0% underwent bariatric surgery (872,499/6,214,682) and 86.0% did not undergo bariatric surgery (5,432,183/6,214,682). Nine studies reported follow-up data, and the mean follow-up was 5.1 years (range: 0–20 years). Five studies reported patient age, and the mean age of included patients was 49.8 years (Table 2).

**Table 2:** Colorectal cancer data for included obese patients

Author	Number	Follow-up in years (range)	Mean age (Years)	Number Surgery	Number No Surgery	CRC Surgery	CRC No Surgery
Adams	16,037	12.5	-	6595	9442	25	52
Aravani	1,002,607	3.0 (1–16)	-	39,747	962,860	43	3237
Bailly	1,045,348	5.3	57.3	74,131	971,217	423	12,629
Ciccioriccio	20,571	4.3 (5–10)	43.6	20,571	-	22	-
Gomez	1,837,594	3.5	53.9	247,015	1,590,579	3540	29,822
Khalid	28,908	5.0	-	19,272	9636	66	55
Lazzati	1,140,347	5.7	49	288,604	851,743	329	4434
Rustgi	98,090	-	-	33,435	64.655	-	-
Schauer	88,625	3.5	45.1	22.198	66.427	-	-
Tao	542,358	4.2 (0–20)	-	49,931	492,427	155	3158
Tsui	394,197	-	-	71,000	323,197	240	1334
	6,214,682	5.1 years	49.8	872,499	5,342,183	4843	54,721

*CRC* colorectal cancer

### Colorectal Cancer Risk following Bariatric Surgery

**Fig. 2:**
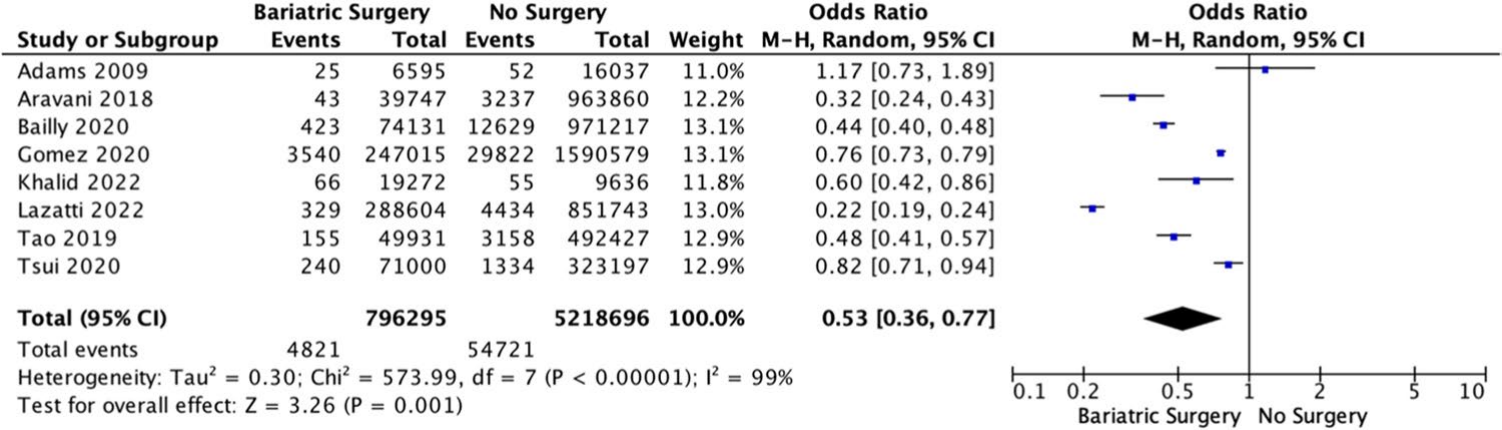
Forest plot illustrating colorectal cancer risk in obese patients who underwent bariatric surgery and those who did not

**Fig. 3:**
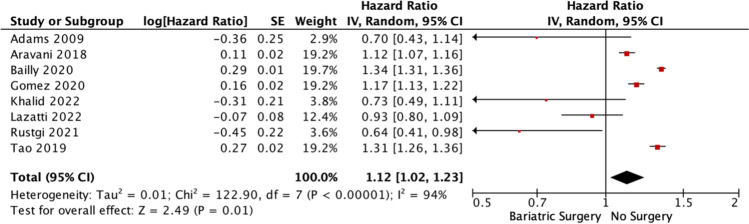
Forest plot illustrating the risk of colorectal cancer in obese patients who underwent bariatric surgery and those who did not using generic inverse variance time-to-effect analysis

Overall, patients with obesity who underwent bariatric surgery were less likely to develop CRC than their counterparts (*P* < 0.001, †). In total, 0.6% of patients with obesity who underwent bariatric surgery later developed CRC (4843/872,499), while 1.0% of patients with obesity who did not undergo bariatric surgery later developed CRC (54,721/5,432,183) (Table 3). Eight studies provided data for inclusion in the meta-analysis [31–33, 35–37, 40, 41]. Patients living with obesity who underwent bariatric surgery were less likely to develop CRC than their counterparts (OR: 0.53, 95% CI: 0.36–0.77, *P* < 0.001, *I*^2^ = 99%) (Fig. 2). Eight studies used time to effect modeling to establish CRC risk following bariatric surgery [31-33, 35–38, 40]. In this analysis, patients with obesity who did not undergo bariatric surgery were more likely to develop CRC than their counterparts who underwent bariatric surgery (OR: 1.12, 95% CI: 1.02–1.23, *P* < 0.001, *I*^2^ = 94%) (Fig. 3).

**Table 3:** Risk of developing colorectal cancer in those who underwent bariatric surgery and in unoperated patients

	Bariatric surgery	No surgery	*P* value
Colorectal cancer	4,843 (0.6%)	54,721 (1.0%)	<0.001*, †
No colorectal cancer	867,656 (99.4%)	5,377,462 (99.0%)	

*Denotes statistical significance

†Denotes Fisher’s exact test

### Colorectal Cancer Risk by Bariatric Surgery

Four studies provided data for inclusion in NMA [33, 34, 36, 41]. These included data in relation to 1,476,151 patients with obesity, of whom 88.3% did not undergo surgery, 2.5% underwent LAGB (35,247/1,476,151), 4.6% underwent SG (67,387/1,476,151), and 4.6% underwent GB (68,477/1,476,151).

**Fig. 4:**
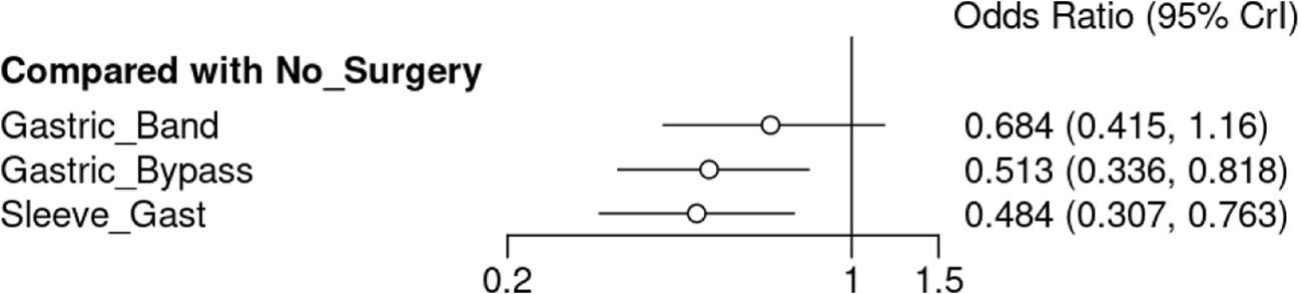
Forest plot comparing the risk of colorectal cancer in obese patients who underwent gastric banding, gastric bypass, sleeve gastrectomy, and no previous bariatric surgery

**Fig. 5:**
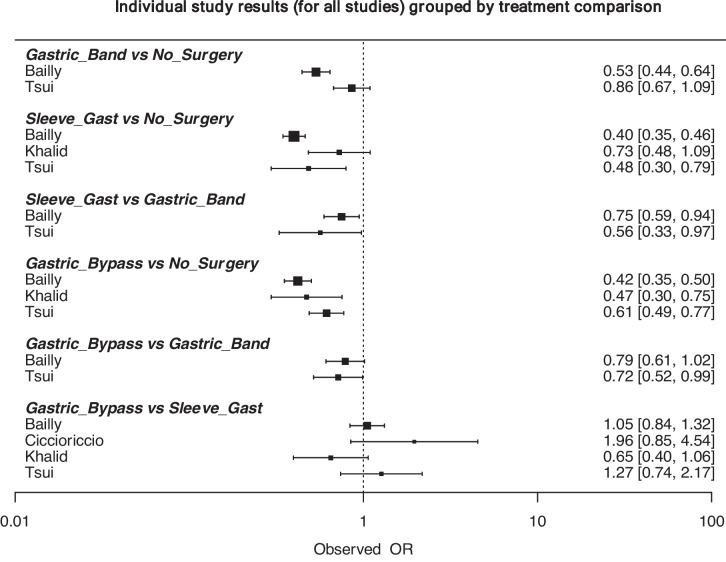
Data summary for all individual studies included in the network meta-analysis assessing colorectal cancer for each surgical approach (represented as odds ratios and 95% confidence intervals)

**Fig. 6:**
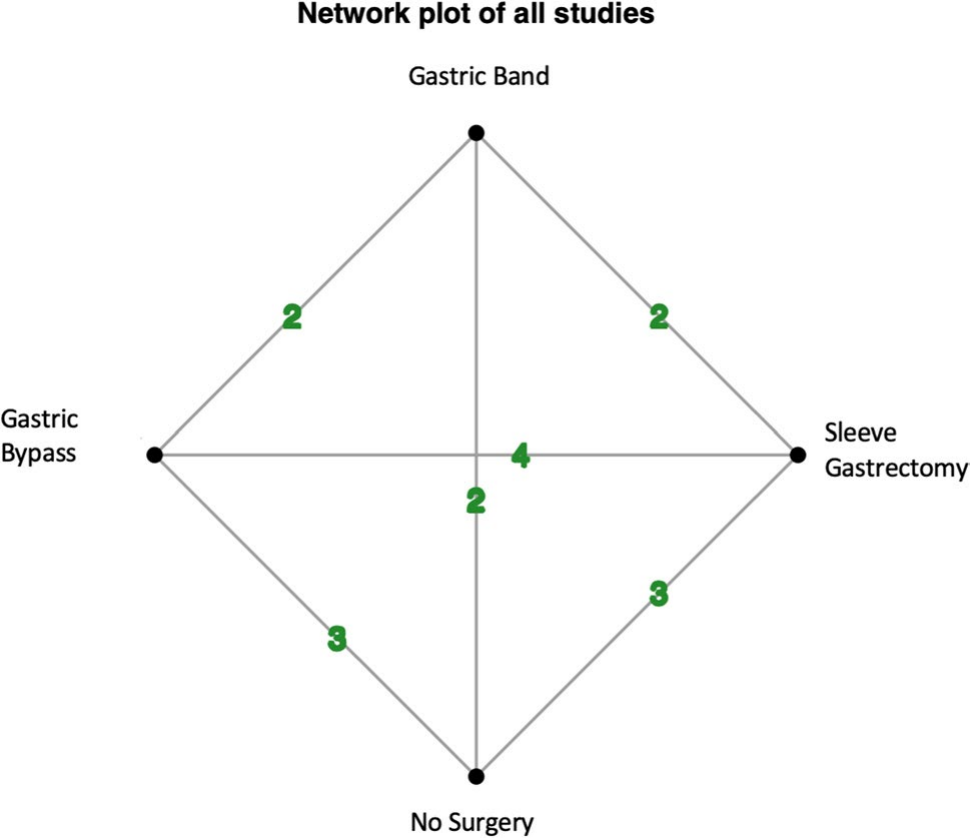
Network plot for all individual studies included in the network meta-analysis

Of those who did not undergo surgery, 1.1% developed CRC (14,018/1,304,050). Of those who underwent LAGB, 0.5% developed CRC (185/36,237). Of those who underwent SG, 0.4% developed CRC (253/67,387). Of those who underwent GB, 0.3% developed CRC (236/68,477) (*P* < 0.001, *χ*^2^) (Table 4). At NMA, patients with obesity undergoing GB (OR: 0.513, 95% CI: 0.336–0.818) and SG (OR: 0.484, 95% CI: 0.307–0.763) were less likely to develop CRC than those who did not undergo bariatric surgery (Fig. 4). Overall study results and the associated network plot are outlined in Figs. 5 and 6. The ranking table for these analyses is illustrated in Table 5.

**Table 4:** Risk of developing colorectal cancer based on bariatric surgery type and in unoperated patients

	No Surgery	LAGB	SG	GBS	*P* value
Colorectal Cancer	14,018 (1.1%)	185 (0.5%)	253 (0.4%)	236 (0.3%)	<0.001*, χ^2^
No Colorectal Cancer	1,290,032 (98.9%)	36,052 (99.5%)	67,134 (99.6%)	68,241 (99.7%)	

*LAGB* laparoscopic gastric band, *SG* sleeve gastrectomy, *GBS* gastric bypass surgery

*Denotes statistical significance

χ^2^Denotes chi-square test

**Table 5:** Ranking table for the bariatric surgery approaches and their impact on colorectal cancer risk

-	Rank 1	Rank 2	Rank 3	Rank 4
Sleeve gastrectomy	0.5987625	0.34635	0.0531	0.0017875
Bypass surgery	0.358525	0.5490125	0.0885375	0.003925
Laparoscopic gastric banding	0.0418875	0.100325	0.796525	0.0612625
No surgery	0.000825	0.0043125	0.0618375	0.933025

### Risk of Bias Assessment

There was low-to-moderate risk of bias among the included studies: Overall, 9 of the included studies had low risk of bias (81.8%) [31-36, 39, 40], while just 2 of the studies included illustrated some concerns for bias (18.2%) [38, 41]. None of the included studies were concerned to have a high risk of bias. Risk of bias assessment using the Newcastle Ottawa Scale is demonstrated in Table 1.

## Discussion

An almost 50% relative reduction in CRC was observed in those who underwent bariatric surgery at a mean follow-up of 5 years. This analysis included data from 11 independent registries, which encompassed 6.2 million patients with obesity. The most important finding in this study was the data supporting the role of bariatric surgery in reducing the risk of developing CRC in patients with obesity, supporting the work of previous authors [23, 24, 42]. The results illustrate the potentially beneficial implications of bariatric surgery at a population level in reducing CRC development. At present, there is a lack of awareness regarding the relationship between obesity and the risk of developing cancers. Most patients are unaware that obesity affects their cancer risk [43–45], despite being aware of an increased risk of cardiovascular and diabetic complications [46]. When informed of this causative link, most patients are willing to attempt weight loss interventions, including bariatric surgery [43, 45]. Thus, discussing the potentially protective role of bariatric surgery in reducing future CRC risk, among other obesity-related malignancies and diseases, could be incorporated into preoperative counseling for patients suitable to undergo bariatric surgery [47–51].

In this study, people with obesity who underwent bariatric surgery had a reduced incidence of CRC relative to their counterparts who did not undergo surgery. Overall, the absolute risk of developing subsequent CRC was reduced to 0.4% (0.6%, 4,843/872,499 vs. 1.0%, 54,721/5,432,183), as well as an estimated relative reduction of 47% observed in this analysis of over 6.2 million people with obesity (OR: 0.53, 95% CI: 0.36–0.77). These findings support the data provided by previous meta-analyses: In their analysis of over 1.2 million patients, Almazedi et al. illustrated a 35% reduction in CRC risk for people with obesity who underwent bariatric surgery [24]. Afshar et al. previously described a reduced risk of CRC following bariatric surgery in their meta-analysis, which included over 100,000 patients with obesity (OR: 0.73, 95% CI: 0.59–0.90) [23]. While patients with obesity have a greater propensity to develop cancers such as CRC [52–54], the multidisciplinary team is further challenged in providing equitable treatment and care for these patients due to the added complexity of healthcare provision for patients with obesity [55]. Therefore, managing patients with obesity present an imminent challenge for healthcare services in ensuring patient outcomes are optimized for this unique and increasingly prevalent patient subgroup [3–5].

Interestingly, patients with obesity who underwent laparoscopic sleeve gastrectomy (OR: 0.484, 95% CI: 0.307–0.763) or laparoscopic gastric bypass (OR: 0.513, 95% CI: 0.336–0.818) were significantly less likely to develop CRC than those who did not undergo bariatric surgery. A laparoscopic gastric bypass is a more technically challenging procedure associated with slightly increased morbidity and mortality rates; however, long-term outcomes are similar. [56]. While implications of such bariatric surgeries on oncological outcomes (such as CRC risk) are important, the primary rationale is to improve patient health and quality of life. Several contemporary, robust analyses of high-quality evidence have evaluated the implications of bariatric procedures on human health: Kang et al. reported on a NMA of randomized controlled trials (RCTs) where the mean BMI reduction observed for SG were 14.4 kg/m^2^ (*n* = 257), 13.5 kg/m^2^ for GB (*n* = 355), and 10.6 kg/m^2^ for LAGB (*n* = 153) [57]. Ding et al. performed an NMA of RCTs which illustrated the value of both GB and SG for maintaining long-term remission of type 2 diabetes mellitus relative to LAGB [58]. Interestingly, some studies have linked bariatric surgery to an increased risk of CRC: In their analysis of 77,111 patients with obesity, Derogar et al. observed an increase in CRC incidence in patients with obesity who underwent bariatric surgery over time (OR: 1.60, 95% CI: 1.25–2.02), which doubled 10 years following surgery (OR: 2.00, 95% CI: 1.48–2.64) [59]. Similarly, Ostlund et al. analyzed the Swedish cancer registry data to establish an increased risk of CRC following bariatric surgery (OR: 1.52, 95% CI: .06–2.11) [60]. These results are refuted by the data of the current analysis. Thus, while SG and GB improve CRC risk relative to LAGB and no surgery, pre-operative counseling and decision-making should include the important medical and oncological data reported by these previous authors in conjunction with the current analysis results.

While data from Memorial Sloan Kettering and MD Anderson Cancer Centre illustrate no compromise in short-term surgical outcomes following CRC resection [61, 62], the data illustrating the contributary role of obesity to CRC development is irrefutable [10-12]. Obesity is associated with a state of chronic inflammation and immune paralysis [63], which is correlated directly with an increased risk of malignancy [64, 65]. There is excessive availability of macronutrients in adipocytes which stimulates an endogenous release of inflammatory markers (such as tumor necrosis factor-alpha (TNF-a) and interleukin-6 (Il-6)) while reducing adiponectin production, ultimately inducing a pro-inflammatory state of excessive oxidative stress [64, 66]. Excessive inflammation within cancer cells and the associated local stromal and inflammatory cells promote oncogenesis, leading to a pro-inflammatory tumor microenvironment [65, 67], which promotes oncogenesis at all stages. Furthermore, increased insulin and insulin-like growth factor-1 (IGF-1) concentrations in the circulation of humans have been illustrated to independently predict malignancy incidence [68]. This reported correlation and cancer incidence are likely due to the direct effect of such hormones on the receptors of the external cellular surface of specific cancer cells, which then act as downstream mediators in the canonical insulin signaling cascade within these cells [69], promoting tumor development. Notwithstanding the long-term durability of bariatric surgery in facilitating weight loss and metabolic improvement [70], it is plausible that there may be secondary implications for reducing CRC risk.

The current study has several limitations. Firstly, the data used in this analysis was obtained from regional, national, and international registries, rendering the data subject to the inherent limitations associated with relying on registry data [71], and relying on a moderate level of evidence to provide informative results into a complex healthcare concern. Secondly, patients included in this study span four decades. During this period, we have observed the evolution in bariatric surgical practice and a significant increase in trends in the obesity and CRC incidence, as well as the indications for bariatric surgery [16, 72, 73]. Finally, the current analysis falls short of addressing the impact of bariatric surgery on reducing CRC within subgroup populations, including those based on age, gender, and ethnicity, while lacking long-term follow-up data, including cancer survival and recurrence. Nevertheless, the authors wish to highlight that the current meta-analysis of over 6.2 million patients further establishes the clinical role of bariatric surgery in reducing CRC risk in patients with obesity at a population level.

In conclusion, the current systematic review and meta-analysis illustrate the potential role of bariatric surgery in reducing future CRC in patients with obesity at a population level. GB and SG provide the greatest reduction in CRC risk. Patient counseling and shared decision-making ab initio of their bariatric surgery should include this data on CRC incidence in conjunction with the other important health implications of bariatric surgery.
